# Moyamoya: A Rare Cause of Cerebral Vascular Accident

**DOI:** 10.5811/cpcem.2017.2.32483

**Published:** 2017-05-24

**Authors:** Brian Merritt, Matt Pitzer, Robyn King, John Ashurst

**Affiliations:** * Lake Erie College of Osteopathic Medicine, Erie, Pennsylvania; † Duke LifePoint Conemaugh Memorial Medical Center, Department of Emergency Medicine, Johnstown, Pennsylvania

## CASE REPORT

**Image 1:**
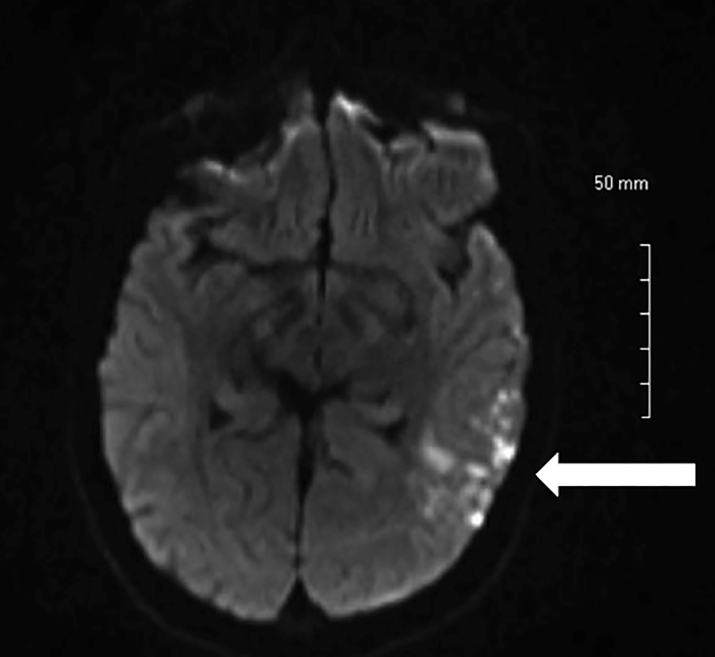
MRI of the brain showing multiple foci of subacute infarcts. *MRI,* Magnetic resonance imaging

**Image 2:**
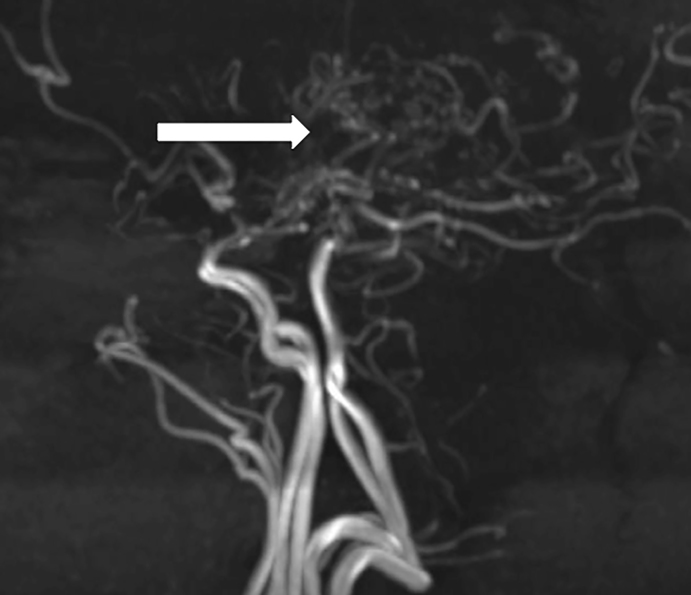
MRA of the brain depecting the classic “puff of smoke” with associated stenosis involving the A1, M1, and P1 segments bilaterally. *MRA*, Magnetic resonance angiogram

A 20-year-old Caucasian female presented with an altered mental status that began one day prior to arrival. The patient claimed to know the answers to questions, but was only able to reply with the answers of “yes” or “I don’t know.” Her past medical history consisted of aortic insufficiency. Her pulse was 109, with a blood pressure of 144/99. Neurological exam revealed that she had expressive aphasia but no facial droop, localized weakness or sensory deficits. A computed tomography of the head showed no acute intracranial hemorrhage, but did show areas of decreased attenuation within the deep white matter of the left frontal lobe without discernible mass effect. A magnetic resonance image (MRI) of the brain showed multiple foci of subacute infarcts (Image 1), while an MR angiogram (MRA) revealed stenosis involving the A1, M1, and P1 segments bilaterally (Image 2).

## DISCUSSION

Moyamoya disease (MMD) is an occlusive cerebrovascular disease characterized by stenosis of the terminal aspect of the internal carotid artery and an abnormal network of basal vessels.^1^ The etiology of MMD is unknown, but both congenital and acquired processes may play a role in its development. The incidence of disease is 4.6 times higher in Asian Americans as compared to their Caucasian counterparts and shows a predominance for females.^1^ Catheter angiography is the gold standard for diagnosis, but due to its invasive nature MRA has gained in popularity. On MRA, the classic “puff of smoke” may be visualized due to collateral vessel formation after arterial stenosis. Patients who are conservatively managed experience stroke at a rate of 3.2%–15.0% annually.^2^ In those who underwent postoperative direct revascularization, the annual stroke rate decreased to 0.0%–1.6%, while those who had undergone postoperative indirect revascularization presented an annual stroke rate of 0%–14.3% annually.^2^

CPC-EM Capsule

What do we already know about this clinical entity?

Moyamoya is a rare clinical entity characterized by stenosis of the internal carotid artery causing those afflicted to have stroke like symptoms.

What is the major impact of the image(s)?

The image depicts the classic “puff of smoke” seen in those with Moyamoya.

How might this improve emergency medicine practice?

Although classically seen in those with Asian descent, the emergency physician must be aware of the diagnosis in all patient populations.

## References

[1] Kim JS (2016). Moyamoya disease: epidemiology, clinical features, and diagnosis. J Stroke.

[2] Kim T, Oh CW, Band JS (2016). Moyamoya disease: treatment and outcomes. J Stroke.

